# Surgical Extirpation of Glomus Tumor from Rare Localization on the Upper Extremity

**DOI:** 10.1155/2013/570945

**Published:** 2013-09-25

**Authors:** Jan Hrubý, Robert Novotný, Miroslav Špaček, Petr Mitáš, Jaroslav Hlubocký, David Janák, Ctibor Povýšil, Jaroslav Lindner

**Affiliations:** ^1^2nd Department of Cardiovascular Surgery, General Teaching Hospital, Prague and 1st Faculty of Medicine, Charles University, U Nnemocnice 2, 128 08 Prague 2, Czech Republic; ^2^Department of Pathology, General Teaching Hospital, Prague and 1st Faculty of Medicine, Charles University, U Nemocnice 2, Prague 2, Czech Republic

## Abstract

*Objective*. To report on a very rare case of a glomus tumor manifested on the upper arm in a healthy young male patient. *Case Presentation and Intervention*. A 22-year-old male patient presented with bluish multifocal venous malformation on the left upper arm and was admitted for venous malformation excision. Pain, discomfort, and upper arm paraesthesia had been present for almost 6 years. Ultrasonography revealed septet tumor without blood flow in the subcutaneous region of anterior aspect of the upper arm. A multifocal venous malformation approximately 5–10 mm in diameter was excised. Histological examination showed dilated vascular area with proliferated glomus cells with round nucleus in the wall of dilated vascular structures. Based on histological examination, the final diagnosis was made as “glomangioma.” *Conclusion*. Histological examination is the only method that can establish final diagnosis. Currently, the only available treatment for this type of tumor is surgical excision.

## 1. Introduction

 Glomus tumors are rare vascular lesions representing approximately 1% of all hand tumors [1]. First clinical description of glomus tumor is dated back to 1924 and was published by Moor et al. [2]. Typical manifestation of these tumors is in young individuals within the age of 20–40 with no sex predilection, except in the subungual lesions which are far more common in women [3]. They are mainly found at the tip of digits and are presented by classic triad: severe pain, point tenderness, and cold sensitivity. Clinical features include blue discoloration, palpable nodule, and nail deformity in subungual tumors. From histological point of view, glomus tumor and its variants are benign neoplasms requiring only a simple excision as a definite method of treatment. Our case report presents a lesion that was superficial and it was not related to any major blood vessels. Only a couple of cases of extradigital glomus tumors have been found in the upper arm region and have been published (the latest [4]).

## 2. Clinical Presentation and Intervention

**Figure 1:**
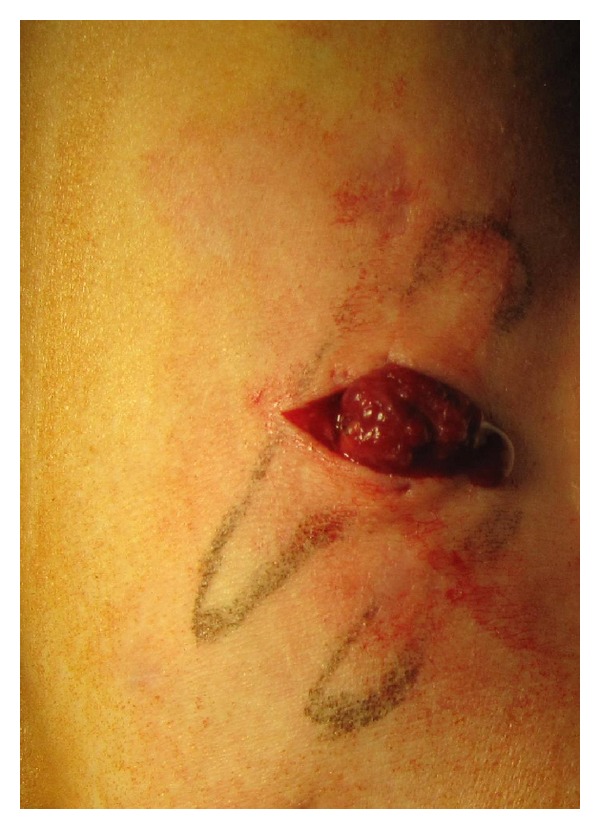
Perioperative finding.

**Figure 2:**
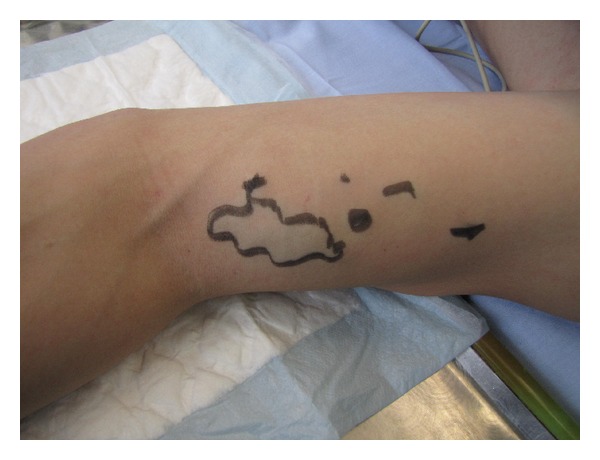
Multifocal location—preoperative finding.

**Figure 3:**
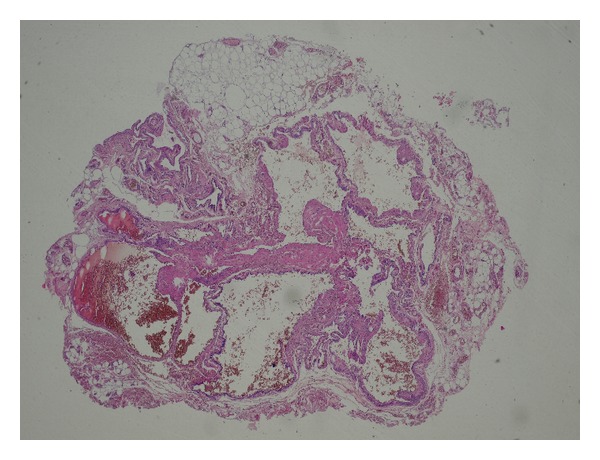
Tumour histology—dilated vascular areas.

**Figure 4:**
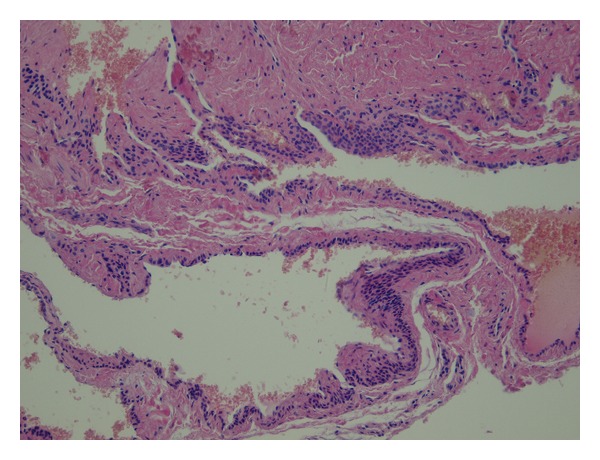
Detail—proliferated glomus cells with round nuclei in the wall of dilated vascular structures.

 A 22-year-old patient was admitted to the hospital for the excision of painful multifocal vascular deformity on the left upper arm (Figure 1), which was clinically similar to varicose vein malformation. Besides the presence of venous malformation on the left upper arm, the patient was healthy without any additional pathology found during general patient examination upon admission to the hospital. Patient's medical history was insignificant. Pain, discomfort, and upper arm paraesthesia had been present for almost 6 years. When patient performed physical work with his left arm, pain and paraesthesia would occur on the anterior and posterior region of the shoulder and anterior region of the arm, as well as the left pectoral region. Examination performed by ultrasonography described septet tumor without blood flow in the subcutaneous region of the anterior aspect of the upper extremity. The tumor presented itself as a bluish multifocal area 5–10 mm in diameter, just above biceps muscle (Figure 1). During surgery, the tumor was exposed through a short skin incision on the left upper arm (Figure 2). The operation was followed by permanent and immediate relief of pain. Histological examination showed dilated vascular area with proliferated glomus cells with a round nucleus in the wall of dilated vascular structures (Figures 3 and 4). Final diagnosis, based on the histological examination, was glomangioma.

## 3. Discussion

 The glomus tumors are classified as a solitary glomus tumor, glomangioma, or nonchromaffin paraganglioma. Glomangiomas are rare, mainly benign blue-red painful tumors. These tumors account for 1% of all arm soft tissue tumors [1]. They belong to a group of tumors called hemangioma. Glomus body is a specialized form of arteriovenous anastomosis which regulates heat [5]. It is located in the *stratum reticularis* of dermis, and it is most frequently encountered in the subungual region, lateral areas of the digits and palm. The glomus body is made up of an afferent arteriole. This arteriole is derived from the small arterioles that supply dermis. The arteriole branches into two or four preglomic arterioles. These arterioles are endowed with the usual complement of the muscle cells and an internal elastic lamina. They blend gradually into a thick-walled segment well knows as the Sucquet-Hoyer canal. The entire glomic complex is surrounded by a collagenous tissue which is composed of vessels and small nerves [5].

 They are generally manifested as a solitary lesion. In some cases we have seen multiple lesions. The lesions develop as small blue-red nodules and are usually located in the deep dermis or the subcutis of the upper or the lower extremity. The most common location where this tumor presents itself is the subungual region of the finger. Also, extradigital sites have been noted. There are only few extra digital tumors documented on the upper extremity (the latest [4]). Other anatomical localizations where this tumor was found include urinary bladder, small intestine, stomach, larynx, and other unusual anatomical locations [6–9]. Tumors located superficially are often presented with paroxysms of pain which radiate away from the lesion. These symptoms are often exacerbated by changes in the temperature, especially the exposure to cold. The deeply seated glomus tumors are not presented with commonly related symptoms [3].

 Extradigital glomus tumors may be encountered in the hand and in the forearm. Surgeons should be aware of this possibility, and they should consider it in the differential diagnoses of such vascular lesions of the upper extremity [10].
